# Do self-report and medical record comorbidity data predict longitudinal functional capacity and quality of life health outcomes similarly?

**DOI:** 10.1186/1472-6963-12-398

**Published:** 2012-11-14

**Authors:** Adesuwa B Olomu, William D Corser, Manfred Stommel, Yan Xie, Margaret Holmes-Rovner

**Affiliations:** 1 College of Human Medicine, Clinical Center Building, Michigan State University, 788 Service Road, Room B329, East Lansing, MI, 48824, USA; 2 College of Human Medicine, Institute for Health Care Studies, Michigan State University, 965 Fee Road, Room A134, East Lansing, MI, 48824, USA; 3 College of Nursing, Michigan State University, 424A West Fee Hall, East Lansing, MI, 48824, USA; 4 PharmNet/i3, 5572 Star Flower Dr, Haslett, MI, 48840, USA; 5 Health Services Research, Michigan State University, C203 E. Fee Hall, East Lansing, MI, 48824, USA

**Keywords:** Comorbidity, Self-report, Medical record data, Functional capacity, Quality of life

## Abstract

**Background:**

The search for a reliable, valid and cost-effective comorbidity risk adjustment method for outcomes research continues to be a challenge. The most widely used tool, the Charlson Comorbidity Index (CCI) is limited due to frequent missing data in medical records and administrative data. Patient self-report data has the potential to be more complete but has not been widely used. The purpose of this study was to evaluate the performance of the Self-Administered Comorbidity Questionnaire (SCQ) to predict functional capacity, quality of life (QOL) health outcomes compared to CCI medical records data.

**Method:**

An SCQ-score was generated from patient interview, and the CCI score was generated by medical record review for 525 patients hospitalized for Acute Coronary Syndrome (ACS) at baseline, three months and eight months post-discharge. Linear regression models assessed the extent to which there were differences in the ability of comorbidity measures to predict functional capacity (Activity Status Index [ASI] scores) and quality of life (EuroQOL 5D [EQ5D] scores).

**Results:**

The CCI (R^2^ = 0.245; p = 0.132) did not predict quality of life scores while the SCQ self-report method (R^2^ = 0.265; p < 0.0005) predicted the EQ5D scores. However, the CCI was almost as good as the SCQ for predicting the ASI scores at three and six months and performed slightly better in predicting ASI at eight-month follow up (R^2^ = 0.370; p < 0.0005 vs. R^2^ = 0.358; p < 0.0005) respectively. Only age, gender, family income and Center for Epidemiologic Studies-Depression (CESD) scores showed significant association with both measures in predicting QOL and functional capacity.

**Conclusions:**

Although our model R-squares were fairly low, these results show that the self-report SCQ index is a good alternative method to predict QOL health outcomes when compared to a CCI medical record score. Both measures predicted physical functioning similarly. This suggests that patient self-reported comorbidity data can be used for predicting physical functional capacity and QOL and can serve as a reliable risk adjustment measure. Self-report comorbidity data may provide a cost-effective alternative method for risk adjustment in clinical research, health policy and organizational improvement analyses.

**Trial registration:**

Clinical Trials.gov NCT00416026

## Background

Comorbidity is an important independent predictor of in-hospital mortality and is routinely used in risk adjustment. [1-4] The influence of comorbidity on patient outcomes such as quality of life and functional status is less well known. [4-7] As part of a larger study of chronic disease self-management over eight months post-hospitalization for acute coronary syndrome (ACS), we sought to evaluate a self report comorbidity measure that would minimize the need to access medical records while maintaining predictive validity as reflected in the association with functional capacity and quality of life. Evaluating interventions in patient populations with chronic conditions frequently requires attention to comorbidity risk adjustment when examining global outcome measures such as functional capacity, health status and emotional well-being [2].

Although several comorbidity measures have been developed [6-9] the Charlson Comorbidity Index (CCI) derived from medical record data remains the most widely used risk adjustment tool. [10,11] Charlson et al [10] initially used multivariate analysis to develop a weighted comorbidity index designed to predict one-year mortality. This index was initially tested on a sample of medical hospital inpatients and later validated on a test population of breast cancer patients at another acute care facility. [10] The CCI has also been adapted for use with administrative data as a predictor of mortality, length of stay, resource utilization, and treatment complications [12].

Comorbidity information from either medical records or administrative data sources can be limited by a) quality of documentation, [13,14] b) limited availability of recent documentation, and c) under-reporting of pre-admission conditions judged by clinicians to be less pertinent to patients’ admitting diagnoses [15-17]. Medical record notes may also frequently contain elements of both patient self-report and earlier professional documentation, sometimes offering a hybrid source of original data [15,16,18].

These limitations have led to increased interest in using patient self-report to calculate composite comorbidity levels [14,17,19-21]. Research has shown that many patients can accurately report their current [17,21-23] and past medical conditions, [24-26] including comorbidities. [14,21,27] However, data are limited regarding how well a self-report comorbidity score predicts functional capacity or quality of life health outcomes [4].

The few global self-report comorbidity measures have been far less widely used and validated than administrative or medical record derived measures. While self-report measures are subject to missing data due to incomplete filling of forms, the main challenge with any self-report measure is to translate medical language to plain language that patients can understand without assistance from a health professional. The most frequently used measure was developed by Katz et al [14]. The Self-Administered Comorbidity Questionnaire (SCQ) was designed to be an equivalent self-report analog of the Charlson Index. Silliman et al [28] designed a “quasi” self-report study using time to death as the outcome of interest but only used the SCQ measure with only 303 breast cancer patients. Other self-report measures are generally disease specific rather than general measures. The SCQ was therefore chosen for the present analyses since it contains the same elements as the CCI and has demonstrated adequate face validity due to its careful development.

The objective of this study was to evaluate the performance of the SCQ to predict functional capacity, quality of life health outcomes compared to CCI medical records data in the same patients, admitted to hospital with a diagnosis of ACS.

## Methods

### Sample

Medical record data were collected from a sample of 719 ACS patients hospitalized in five mid-Michigan hospitals as part of a trial of a telephone intervention program. The intervention was designed to improve patients’ health behaviors within a post-discharge quality improvement program. [29] For detailed information, see Holmes-Rovner et al [29].

Data concerning patients’ socio-demographic characteristics, comorbid conditions, and subsequent functional capacity and quality of life were collected from: 1) medical record chart abstractions at baseline, as well as 2) structured panel telephone patient interviews concerning patients’ functional capacity and quality of life at approximately three and eight months after discharge.

All medical records data were collected by a group of nurse chart abstractors who were trained by the study Community Project Manager (CPM). Each chart abstractor used a standard data collection sheet and made reference to a chart abstraction manual concerning specific data field definitions and parameters.

Telephone interviews of post-discharge patient health outcomes were conducted by trained survey researchers at the authors’ university-based survey institute at three time periods: 1) at baseline shortly after index hospitalization (Mean = 14.11 days, SD = 9.6), 2) at three months, and 3) eight months after index hospitalization. The study was approved by Michigan State University Biomedical, Health Sciences IRB.

Of 719 consenting subjects, 525 (73.0%) patients completed a baseline interview and had complete data concerning their personal characteristics and comorbid conditions using both the CCI and SCQ methods. A total of 440 (83.9% of baseline interviewed) completed a three-month interview, and 388 (88.2% of three-month interviewed) completed an eight-month interview. Including attrition from initial consent, 72.5% completed a baseline interview, with further panel attrition of 16.6% and 11.8% at three and eight months, respectively. Before any data were collected, approval had been obtained from the authors’ institutional review board and each participating hospital. In order to ensure largely complete information, we reduced the effective analytic sample sizes to 525 (baseline), 438 (3-month) and 387 (8-month).

### Study measures

Patients’ socio-demographic characteristics included: race (White and Non-White), marital status category (married and divorced/separated/widowed), education completed (less than high school and high school or greater), and family income level category (< $15,000 per year, and $15,000 or more per year). Patient age (in complete years) was treated as a continuous variable.

Depressive symptoms were measured using the Center for Epidemiologic Studies-Depression Scale (CESD). [30] The CESD [30] is a validated 20-item self-report screening instrument designed to measure the frequency/duration of depressive symptoms in non-psychiatric community populations. A composite CESD score of 16 or greater is correlated with a mild to moderate level of depressive symptoms and was used as a cut-point in our study [30-32]. The instrument has demonstrated adequate internal consistency (α = .85 to .95) in general, psychiatric, and medical populations [30].

Medical records were used to generate the CCI composite comorbidity scores [10,11]. The CCI has been tested with large samples in numerous settings. [1,6,7,33] With this index, a patient’s composite CCI score is calculated as a weighted sum of the presence of 19 documented health conditions such as Congestive Heart Failure, Diabetes, or Peripheral Vascular Disease. For our analyses, we used the original Charlson weighting scheme assigning a score of 1, 2, 3, or 6 to each appropriate comorbidity condition and summing these values.

The Katz Self-Administered Comorbidity Questionnaire (SCQ) was used in its full 19 item version. [14] The SCQ includes items corresponding to each of those in the CCI [10,11]. The individual item weightings for the Katz SCQ [14] method are almost entirely the same as for the CCI. [10,11] In the original Katz et al study, test-retest reliability, assessed with the intra-class correlation coefficient, was 0.91 for the questionnaire and 0.92 for the CCI. The Spearman correlation between these two measures was 0.63. [14] Subsequent development of the measure has reduced the number of items to thirteen [17].

The outcome measures used in these analyses included the five-item EuroQOL 5D scale, a commonly used Quality of Life measure that combines patient responses at each of the three panel interviews to questions about five quality of life dimensions. [34] These dimensions include Mobility, Self-Care, Usual Activities, Pain/Discomfort, and Anxiety/Depression, with the respondent indicating whether each of these dimensions were frequently, occasionally, or never, a problem in their daily lives.

Functional capacity was measured using the Activity Status Index (ASI). [35] The ASI is a 12-item measure of a person’s physical functional capacity that was developed to correlate with peak oxygen uptake, a criterion particularly relevant to the functional status of cardiac patients. The ASI has been found to have adequate sensitivity to clinical changes and to be moderately (r = 0.58) correlated with other measures of cardiovascular fitness such as measures of peak oxygen intake during exercise testing. [35] Scale scores can range from 0 to 58.2 with higher scores indicating better functional capacity [35].

Information on overall level of cardiac functioning of the ACS patients was included in predictive models, using cardiac ejection fraction, a continuous measure with a possible range of 0% or 100% [36].

### Data analyses

Sample descriptive statistics, including frequencies, percentages, means and standard deviations were calculated to summarize the socio-demographic and health status characteristics of the patients. Mean scores for the EQ5D and the ASI were calculated for the analytic samples including (1) all cases with available information, and (2) the subset with complete information at all three interview times, to gauge the effects of attrition on mean changes over time.

To assess the extent to which there are differences in the ability of the survey-based SCQ [14] and the medical-record-based CCI [10] to predict physical functional capacity (ASI scores) [35] and quality of life (EQ5D scores) [34], linear regression models were used two times: with 3-month, and 8-month ASI and EQ5D outcomes. The initial full models with either ASI scores or EQ5D scores as outcome variables include the following set of covariates/predictor variables: time of interview, age at MI, education, income, gender, race, marital status, depression, ejection fraction at hospitalization, and performance of interventions [percutaneous coronary intervention (PCI) and coronary artery bypass graft (CABG)].

In addition, these models were run twice, either including the SCQ or CCI as independent predictors. A backward stepwise regression approach was employed to eliminate all independent variables, for which the incremental F-test was not significant (p > 0.05) in both models. Comparison of contributions of SCQ and CCI comorbidity information to ASI scores (Table 1) and EQD-5 scores (Table 2) at 3-months and 8-months post-discharge were determined. All data analysis was carried out using the Stata 10.1 software [37].

**Table 1:** **Comparison of contributions of Katz** (**Self**-**Report**) **and Charlson** (**Medical**-**Record**) **comorbidity information to ASI** (**Functional**-**Capacity**) **scores**

**Outcome:3**-**Month ASI** (**N**=**438**)	**Model R**-**Squared**	**R**-**squared Change**	**F**-**Test**/ **F**-**change**	**Degrees of freedom**	**P**-**value**
Model with covariates only^a^	0.317	0.317	14.43	14/423	<0.0005
Covariate model +Katz	0.340	0.023	14.20	1/422	<0.0005
Covariate Model + Charlson	0.331	0.014	14.29	1/422	<0.0035
**Outcome: 8-Month ASI (N=387)**	**Model R-Squared**	**R-squared Change**	**F-Test/ F-change**	**Degrees of freedom**	**P-value**
Model with covariates only^a^	0.334	0.334	13.31	14/372	<0.0005
Covariate model +Katz	0.358	0.025	14.22	1/371	<0.0005
Covariate Model + Charlson	0.370	0.036	21.15	1/371	<0.0005

a. covariates: age, gender, race, education, household income, marital status, depressive symptomatology (CESD), ejection fraction, invasive cardiac procedures.

**Table 2:** **Comparison of contributions of Katz** (**Self**-**Report**) **and Charlson** (**Medical**-**Record**) **comorbidity information to EQD**-**5** (**Quality**-**of**-**Life**) **scores**

**Outcome:3**-**Month EQD**-**5** (**N**=**438**)	**Model R**-**Squared**	**R**-**squared Change**	**F**-**Test**/ **F**-**change**	**Degrees of freedom**	**P**-**value**
Model with covariates only^a^	0.259	0.259	10.56	14/423	<0.0005
Covariate model +Katz	0.288	0.029	17.27	1/422	<0.0005
Covariate Model + Charlson	0.262	0.003	1.64	1/422	>0.201
**Outcome: 8-Month EQD-5 (N=387)**	**Model R-Squared**	**R-squared Change**	**F-Test/ F-change**	**Degrees of freedom**	**P-value**
Model with covariates only^a^	0.240	0.240	8.40	14/372	<0.0005
Covariate model +Katz	0.265	0.025	12.52	1/371	<0.0005
Covariate Model + Charlson	0.245	0.005	2.28	1/371	>0.132

a. covariates: age, gender, race, education, household income, marital status, depressive symptomatology (CESD), ejection fraction, invasive cardiac procedures.

## Results

The patient sample was typical of patients hospitalized for ACS (See Table 3).

**Table 3:** **Demographic** &**clinical characteristics of patients at baseline** (**n** = **525**)

**Variable**	**N**	
**Age at admission**	**525**	**M**=**59**.**73** (SD 12.00)
**Gender**	**525**	
***Male***		**334** (63.6%)
Female		**191** (36.4%)
**White**/**Non**-**White Race**	**525**	
White		**443** (84.4%)
Non-White/Multiracial/Other		**82** (15.6%)
**Current Marital Status**	**525**	
Married		**350** (66.7%)
Divorced/Separated/Widowed		**174** (33.1%)
**Work for Pay of Profit**?	**524**	
Yes		**226** (43.0%)
No		**298** (56.8%)
**Completed Education**	**521**	
Less than High School		**99** (18.9%)
High school diploma or higher		**426** (81.8%)
**Family Income**	**467**	
Less than $15,000 per year		**113** (25.0%)
$15,000 or more per year		**354** (75.8%)
**Activity Status Index**^26^ (scale 0 - 54.55)	**525**	**M**=**29**.**56** (SD 17.21)
**CESD Depression**^27^ (scale 0 - 60)	**524**	**M**=**13**.**56** (SD 10.48)
**EuroQol 5D**		**See Table**4
**Ejection Fraction**	**452**	**M**= **50**.**19** (SD 12.93 )

Changes of EQ5D and ASI over time are shown in Table 4. Results showed no changes in the mean quality of life (EQ5D) scores (F = 0.79, p < 0.455) and a decrement in functional capacity (ASI) from pre-hospitalization to three months after hospital discharge, with a small recovery between the 2^nd^ and 3^rd^ interview five months later (F = 8.22, p < 0.001).

**Table 4:** **Mean EQ5D** (**Quality of Life**) **and ASI** (**Physical Functioning**) **scores over time**

(**A**) **EQ5D:**						
**Time:**	**EQ5D** **(including all cases)**			**EQ5D** **(completers of all interviews)**		
	**Mean**	**St. Dev.**	**N**	**Mean**	**St. Dev.**	**N**
Baseline Interview	0.749	0.262	515	0.760	0.257	381
3-month Interview	0.753	0.271	439	0.768	0.259	387
8-month Interview	0.767	0.265	388	0.767	0.265	388
				F=0.79, p<0.455		
**(B) ASI:**						
**Time:**	**ASI** **(including all cases)**			**ASI** **(completers of all interviews)**		
	**Mean**	**St. Dev.**	**N**	**Mean**	**St. Dev.**	**N**
Baseline Interview	29.55	17.20	525	30.46	16.78	388
3-month Interview	25.04	17.15	439	25.56	17.01	387
8-month Interview	26.87	17.74	388	26.87	17.74	388
				F=0.8.22, p<0.001		

Results from the linear regression models comparing the contributions of SCQ and CCI comorbidity information to ASI scores and EQ5D scores at 3-month and 8-month after hospitalization are shown in Tables 1 and 2.

**Figure 1:**
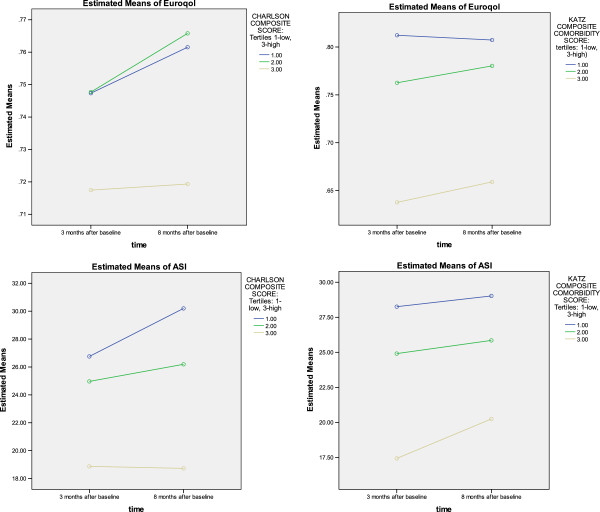
**Comparison of Three and Eight**-**Month CCI and SQC Scores on ASI &EuroQOL Outcomes.** ASI = Activity Status Index, Euroqol = EuroQol5d.

Results revealed that both the SCQ and CCI predicted ASI (functional capacity) at 3-months and 8-months after hospitalization for ACS. The SCQ predicted ASI slightly better at 3-months (R^2^ = 0.340; p < 0.0005 vs. R^2^ = 0.331; p < 0.0035) compared to the CCI. However, the CCI predicted slightly better at the 8-month (R^2^ = 0.370; p < 0.0005 vs. R^2^ = 0.358; p < 0.0005) follow up (See Table 1). It is important to note that the CCI *did not* predict the quality of life (EQD5 scores) at 3-months (R^2^ = 0.262; p > 0.201), or at 8-months (R^2^ = 0.245; p > 0.132) follow-up after ACS. However, the self-report SCQ significantly predicted the EQD5 scores at 3-months (R^2^ = 0.288; p < 0.0005) and 8-months (R^2^ = 0.265; p < 0.0005) follow up (see Table 2). CESD was associated with EQ5D in all analyses with ASI scores as the dependent variable in the regression model with the SCQ measure included, after adjusting for all socio-demographic and other covariates, ASI scores decline steeply after hospitalization (-2.14, p = 0.000) but did not differ significantly between three-months and eight months, suggesting early and stable recovery in functional capacity. Figure 1 graphically depicts these comparative relationships for the two comorbidity measures on both ASI and EQ5D at baseline (lightest lines at bottom) as well at three and eight months after hospital discharge.

Covariates including race, education, marital status, ejection fraction category, and invasive procedure were consistently non-significant influences on both study outcomes when including either SCQ or CCI comorbidity measures. Several sample subgroups including those with higher age, females, and subjects with lower family income levels reported significantly lower levels of functional capacity and quality of life during panel interviews when these terms were included in models with both comorbidity methods.

In summary, the SCQ self-report method was an overall equivalent predictor of both the post hospitalization EQ5D (quality of life) and the ASI (functional capacity) scores compared to the CCI. For both regression models, the incremental and overall R-squared values, albeit fairly low, were slightly higher with the inclusion of the SCQ as predictor instead of the CCI. Similar to at least two earlier studies, [38,39] our fairly low R-squared values may represent the many factors likely impacting the functional capacity and quality of life outcomes for such patients after an acute ACS hospitalization.

## Discussion

In comparing performance of the SCQ and CCI, we found the CCI did not predict the quality of life EQD5 scores after ACS. The CCI was not significantly associated with the EQ5D scores at three months and at eight months after hospitalization for ACS. The SCQ, however, more significantly predicted the EQD5 scores at three-months, and at eight months follow-up. However, CCI is almost as predictive as the SCQ for ASI functional capacity scores at three and eight months, and performing more significantly in predicting quality of life at eight-month follow-up.

Our findings are particularly important because our follow-up period allowed longer post-discharge assessment of functional capacity and quality of life at intervals longer than 30-day mortality, as is most often the case when administrative data are used [9,40,41]. As we reported previously, patients reported more of certain comorbidities using the self-report instrument compared with the medical record [42].

Our results indicate that having additional co-morbidities, as self-reported by patients are relatively strong predictors of quality of life (EQ5D scores); and functional capacity (ASI scores). On average, our analysis revealed that they decline by -0.02 (p < 0.001); and -2.00 (p< 0.001) for each unit increase in the self reports CCI (Katz scores) for the EQ5D and ASI respectively. Similarly, Motl et al [23] found statistically significant inverse associations between the number of self-reported cardiovascular comorbidities and objectively measured and self-reported physical activity. Their study revealed that physical activity levels in persons with muscular sclerosis were associated with the number of self-reported cardiovascular comorbidities, independent of disability status, and other possible cofounding influences. In another smaller study, Bayliss et al [2] found that for certain quality of life assessments, self-reported comorbidity data may provide a more accurate estimate of comorbidity than existing medical record reporting sources. Susser et al [19] compared the predictive validity of self report and administrative CCI using subsequent health services utilization rates and functional decline as outcomes. In contrast to our finding, they found that agreement between self-report and administrative comorbidity data was only poor to fair but both have comparative levels of predictive validity.

Our study also found that higher depressive symptom levels (CESD scores; -0.011, p < 0.001; -0.411, p < 0.001) were significantly associated with both patients’ quality of life and functional capacity. More depressed patients in our sample consistently had lower levels of quality of life and functional capacity after a hospitalization for ACS.

In our multivariate models, certain factors such as age, gender, family income and CESD were significantly associated with the Charlson CCI and self report SCQ in predicting functional capacity. The self-report comorbidity measure more significantly predicted QOL during the eight-month study window.

Male ACS patients generally demonstrated higher QOL and functional capacity levels after discharge. In addition, patients with higher family incomes (> $40,000) did seem to experience both a higher quality of life and level of functional capacity. For future studies, these patterns suggest that research interventions and analyses will need to be effectively targeted to capture differential influences of these factors on many health outcomes.

Katz and colleagues have continued to develop and validate the SCQ in populations of general surgical patients in hospital settings [17]. In addition, a German version of the SCQ, the SCQ-D has recently been developed [21]. The comorbidity measured by the SCQ-D proved to be a valid predictor of the hospitalization and the treatment outcome [21].

There are several limitations of this study. First, the majority of these post discharge data came from a sample of hospitalized patients with ACS from specific community hospitals in the Midwest. Second, the use of hospital medical records may have limited our ability to capture all the patients’ documented conditions as this is affected by the quality of documentation. Wording differences between self-report source (i.e. before your hospitalization) and medical record source (designed to capture both current and past conditions) items may account for some of our observed differences.

## Conclusions

Our results show that the self-report SCQ index is a generally an equivalent predictor of quality of life health outcomes when compared to a CCI score generated from medical records data with both predicting physical functioning similarly. This suggests that patient self-reported comorbidity data can be used for predicting functional capacity and quality of life health outcomes and may serve as a reliable measure for risk adjustment. Since self-report comorbidity data are often more complete than CCI scores based on medical records and can easily be collected from patients in a short survey, they may provide a more cost-effective alternative method for risk adjustment in clinical research, health policy and organizational improvement analyses.

Our results are important in the context of increased use of electronic health records (EHR) and increased use of electronic records data mining. Problem associated with use of administrative data for clinical purposes, including comorbidity measurement are well known. To the extent that administrative EHR data become a source of data to support both clinical and evaluative purposes, they may continue to suffer from problems of accuracy and completeness.

Patient recall of past comorbid conditions in the encounter under time pressure may also be incomplete or inaccurate. In addition, providers frequently use technical language that patients do not understand when inquiring about comorbid conditions. However, with the increased availability of patient portals in EHR systems, our findings suggest that meaningful portal prompts using plain language for patients to directly enter historical or current changes in their comorbid conditions may enable providers and researchers to improve their prediction of longer-term patient outcomes for risk adjustment. Merging the results of such new patient portal surveys with the reports of illnesses in the EHR in real time should support improved patient care and support chronic disease management.

## Abbreviations

ACS: Acute coronary syndrome; AMI: Acute myocardial infarction; CCI: Charlson comorbidity index; SCQ: Self-administered comorbidity questionnaire; QOL: Quality of life; CESD: Center for epidemiologic studies-depression scale; ASI: Activity status index.

## Competing interests

The authors declare that they have no competing interests.

## Authors' contributions

AO; conception and design, drafting and revising of manuscript. WC; design, revising manuscript. MS design, analysis, and interpretation of data, revising manuscript. YX; design and revising of manuscript. MH-R conception and design, revising manuscript. All authors read and approved the final manuscript.

## Authors' information

AO; MD, MS, FACP; Associate Professor of Medicine, Health Services and Outcomes Researcher, College of Human Medicine, Michigan State University, East Lansing MI. WC; PhD, RN,, Research Specialist, Institute for Health Care Studies, College of Human Medicine, Michigan State University, East Lansing MI. MS; PhD, Professor of Statistics, Health Services Research, College of Nursing, Michigan State University, East Lansing, Michigan. YX; PhD, Biostatistician, PharmNet/i3, 5572 Star Flower Dr., Haslett, MI. MH-R; PhD, Professor of Health Services Research, Center for Ethics and Humanities, College of Human Medicine, Michigan State University, East Lansing MI

## Pre-publication history

The pre-publication history for this paper can be accessed here:

http://www.biomedcentral.com/1472-6963/12/398/prepub
